# Enrichment of native plastic‐associated biofilm communities to enhance polyester degrading activity

**DOI:** 10.1111/1462-2920.16466

**Published:** 2023-07-28

**Authors:** Sophie A. Howard, Clodagh M. Carr, Habteab Isaack Sbahtu, Uchechukwu Onwukwe, Maria J. López, Alan D. W. Dobson, Ronan R. McCarthy

**Affiliations:** ^1^ Centre for Inflammation Research and Translational Medicine, Division of Biosciences, Department of Life Sciences, College of Health and Life Sciences Brunel University London Uxbridge UK; ^2^ School of Microbiology University College Cork Cork Ireland; ^3^ SSPC‐SFI Research Centre for Pharmaceuticals University College Cork Cork Ireland; ^4^ Experimental Techniques Centre, College of Engineering, Design and Physical Sciences Brunel University London Uxbridge UK; ^5^ Department of Biology and Geology, CITE II‐B University of Almería, Agrifood Campus of International Excellence ceiA3, CIAIMBITAL Almeria Spain

## Abstract

Plastic pollution is an increasing worldwide problem urgently requiring a solution. While recycling rates are increasing globally, only 9% of all plastic waste has been recycled, and with the cost and limited downstream uses of recycled plastic, an alternative is needed. Here, we found that expanded polystyrene (EPS) promoted high levels of bacterial biofilm formation and sought out environmental EPS waste to characterize these native communities. We demonstrated that the EPS attached communities had limited plastic degrading activity. We then performed a long‐term enrichment experiment where we placed a robust selection pressure on these communities by limiting carbon availability such that the waste plastic was the only carbon source. Seven of the resulting enriched bacterial communities had increased plastic degrading activity compared to the starting bacterial communities. *Pseudomonas stutzeri* was predominantly identified in six of the seven enriched communities as the strongest polyester degrader. Sequencing of one isolate of *P. stutzeri* revealed two putative polyesterases and one putative MHETase. This indicates that waste plastic‐associated biofilms are a source for bacteria that have plastic‐degrading potential, and that this potential can be unlocked through selective pressure and further *in vitro* enrichment experiments, resulting in biodegradative communities that are better than nature.

## Graphical abstract

In this work, we demonstrate an approach to unlock the plastic‐degrading potential of marine plastic‐associated biofilms. We also identify *Pseudomonas stutzeri* as a plastic degrading species and discover three new enzymes potentially linked to polyester degradation.

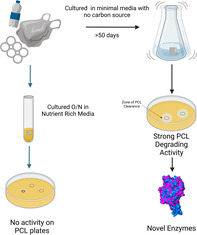

## INTRODUCTION

Plastic pollution is a growing worldwide problem, with 12,000 million metric tonnes (Mt) of plastic waste predicted to be in the environment and landfill by 2050 (Geyer et al., 2017). While recycling can give a second life to some plastic, downstream quality and cost can be limiting factors, resulting in only 9% of plastic waste having ever been recycled (Geyer et al., 2017; Shamsuyeva & Endres, 2021). If plastic is not recycled, it is either sent to landfill where it can pollute the soil and run off pollutes the global water systems, or it is incinerated, potentially releasing toxic fumes, and adding to carbon emissions (Chianga et al., 1992; Jambeck et al., 2015; Li et al., 2001; Royer et al., 2018). A more environmentally friendly alternative is biodegradation of plastic by microorganisms into non‐toxic breakdown products, some of which can be valorised with downstream industrial uses such as succinic acid, which can be used an acidity regulator in the food industry or polyhydroxyalkanoate (PHA), which is a bioplastic (Kenny et al., 2008; Ru et al., 2020).

Multiple species of environmental bacteria have been found to degrade plastic, however many of these do so at a slow rate resulting in only a minor reduction in plastic mass over a period of months. An example of this is an environmental consortia which was shown to be able to decrease polystyrene (PS) weight by ~5% in 6 months (Syranidou et al., 2017). Plastic is not a natural carbon source for these organisms as it is a man‐made substrate which has only been present in their environment for ~100 years, an insufficient amount of time for evolution of completely new enzymes. Many natural properties of plastic make it an undesirable carbon source, such as high crystallinity and high molecular weight, both of which have been linked to poor biodegradation (Albertsson & Karlsson, 1993; Pantani & Sorrentino, 2013; Wei & Zimmermann, 2017). The efficiency of the enzymes that bacteria use for biodegradation is also an issue, likely because there are other preferential substrates available in the environment, so the expression of potential plastic degrading enzymes is not sufficiently induced or optimized to drive meaningful degradation. If we are to make use of bacteria with plastic‐degrading activity, then the efficiency of plastic degradation needs to be enhanced. One strategy to achieve this is to apply selective pressures that drive bacteria and microbial communities to reshuffle their metabolic networks towards the use of plastic as their primary carbon source.

The capacity for biodegradation of plastic by environmental bacteria has recently been linked to levels of plastic pollution, suggesting that humans are driving the evolution of bacteria in the environment to degrade plastic (Zrimec et al., 2021). Zrimec et al. (2021) found that abundance of plastic‐degrading genes was significantly correlated with plastic pollution levels. This suggests that plastic waste would be a good source of bacteria that encode the enzymatic capacity to degrade plastic. Additionally, several plastic‐associated bacterial communities have previously been found to possess plastic‐degrading abilities (Artham et al., 2009; Atiq et al., 2010; Gilan et al., 2004; Morohoshi et al., 2018). Here, we demonstrated that subjecting plastic‐associated native biofilm communities to *in vitro* carbon starvation, where the only source of carbon is plastic, can select for bacteria with putative polyesterases and significantly improved degradation capability of the model polyester substrate, polycaprolactone (PCL).

## METHODS

### Bacterial attachment assay

0.03 g of plastic was sterilized in 70% IMS for 30 min then dried in a laminar flow cabinet. Overnight cultures of *E. coli* BL21 pCOLADuet‐1: *dgcC* (Leech, 2017) were OD_600_ corrected to 0.1 in fresh LB (Miller) supplemented with each sterile plastic and grown at 37°C until OD_600_ ~0.6 was reached, expression of DgcC was induced with 500 μM IPTG and cultures were grown for a total of 24 h. Overnight cultures of *Pseudomonas stutzeri* PS13 were OD_600_ corrected to 0.1 in fresh LB supplemented with each sterile plastic and grown at 30°C for 24 h. Plastic was collected from each culture, gently washed in sterile ddH_2_O 3× to remove unattached bacteria, added to 1 mL sterile PBS then sonicated in Camlab Transsonic T460 bath at 35 kHz for 5 min to remove bacteria attached to the plastic surface. Serial dilutions were performed from the PBS suspension of removed bacteria, spotted in triplicate on LB agar, grown for 16 h at 37°C for *E. coli* and at 30°C for *P. stutzeri*, and colony forming units (CFU) counted. Statistical analysis and graph construction was performed in Prism (version 9.3.1 (350)) throughout.

### Fourier‐transform infrared spectroscopy (FTIR)

FTIR was used to confirm the type of plastic waste collected from the coast of Republic of Ireland (51.8588° N, 8.0020° W). After bacteria were collected from the plastic waste, pieces were dried in a laminar flow hood for 24 h. Based on visual inspection, the plastic waste is expanded PS (EPS), so an EPS box was used as a positive control for FTIR. The spectrum was collected on a Perkin Elmer spotlight FTIR imaging system. It was collected over the range 4000 cm^−1^ to 500 cm^−1^ with a resolution of 4 cm^−1^ using the ATR accessory. The collected spectra were run against the automated NICODOM FTIR Spectra library which gave polystyrene as the closest match for both the EPS control box and environmentally collected sample.

### Collection and enrichment of bacterial populations on plastic waste

Sixteen pieces of EPS plastic waste were cut in half (to approximately 1–2 cm diameter), with one half placed in a 3 mL LB culture and grown overnight with agitation at room temperature, collected for glycerol stock and spotted onto PCL agar plates to assess PCL degradation activity through a zone clearing assay, as described in the following section. The other half was placed into 3 mL high salt M9 minimal media with no carbon source (1× M9 salts (MP Biomedicals), NaCl concentration increased to 35 g/L (the same concentration as sea water), 2 mM MgSO_4_, 0.1 mM CaCl_2_ in autoclaved distilled water, solution filter‐sterilized) and grown for 53 days with agitation at room temperature. After 53 days, the culture was inoculated into 3 mL LB, grown overnight for glycerol stock, and spotted onto PCL agar plates to assess PCL degradation activity through a zone clearing assay.

### PCL zone clearing assay

PCL (Sigma–Aldrich, Mn 80,000) was dissolved in acetone with agitation at 50°C then mixed with LB agar solution (distilled water, 1.5% w/v agar‐agar (Fisher), 2% w/v LB medium (Fisher)) to a concentration of 1% w/v PCL after acetone evaporation (Almeida et al., 2019). This was autoclaved and poured into plates straight after autoclaving to keep PCL in solution, resulting in cloudy agar plates. Overnight cultures of bacteria were grown in LB and OD corrected to OD_600_ 3, 20 μL of this was spotted onto the 1% PCL LBA plates and grown at 30°C for 5 days, with measurements of the diameter of the bacterial spot and the zone of clearance (transparent agar surrounding spot) on days 1, 2, 3 and 5. Diameter of each zone of clearance was measured twice (at 90° angles to each other to account for variation) and the average was used, the diameter of the bacterial spot was subtracted and measurements plotted as stacked bar charts. For single colonies plated on PCL plates, OD_600_ 0.1 was diluted 8 × 10^−4^ and 100 μL was plated and grown for 5 days at 30°C. For supernatant PCL clearance, overnight cultures of bacteria were centrifuged at 4000*g* for 20 min then the supernatant was filtered through 0.2 μm and 60 μL of supernatant was spotted onto 1% PCL LBA plates and incubated at 37°C.

### Metagenomic analysis

Metagenomic DNA was extracted from 5 mL overnight cultures of PS13 original and PS13 enriched communities using the GenEluteTM Bacterial Genomic DNA Kit. Overnight cultures (1% inoculum) were grown at 30°C in LB broth to an OD600 of 1.0–1.5, then cells were pelleted, drained, and stored at −80°C prior to extraction. High molecular weight DNA was obtained, and next‐generation shotgun sequencing was subsequently completed by Eurofins Genomics (Konstanz, Germany) using the Illumina NovaSeq 6000 sequencing system, together with library preparation and initial quality checks. The raw sequence read quality was verified by FastQC (v 0.11.9) within the KBase Predictive Biology platform (Arkin et al., 2018; Zrimec et al., 2021). GOTTCHA2 (v 2.1.7) and Kaiju (v 1.7.2) were employed for taxonomic classification of the metagenomic reads, again facilitated by KBase.

### Strain identification

Genomic DNA was extracted from the isolates using MP Biomedical FastDNA™ Kit. Primers 27F (AGAGTTTGATCMTGGCTCAG) and 1525R (AAGGAGGTGWTCCARCC) (Wawrik et al., 2005) were used in PCR to amplify ~1500 bp of the 16S rRNA gene of the isolates following the standard protocol for Thermo Scientific™ DreamTaq polymerase. Additionally, to further distinguish isolates identified as *P. stutzeri* using 16S rRNA sequencing, primers UP1 (GAAGTCATCATGACCGTTCTGCAYGCNGGNGGNAARTTYGA) and UP2r (AGCAGGGTACGGATGTGCGAGCCRTCNACRTCNGCRTCNGTCAT) (Yamamoto & Harayama, 1995) were used to amplify ~1200 bp of the *gyrB* gene and primers PsEG30F (ATYGAAATCGCCAARCG) and PsEG790R (CGGTTGATKTCCTTGA) (Mulet et al., 2009) were used to amplify ~760 bp of the *rpoD* gene. Qiagen QIAquick PCR Purification Kit was used to purify the constructs, which were sequenced using Source Bioscience sanger sequencing. Genomic DNA isolation, PCR and sequencing were performed twice to reach a consensus sequence for each isolate for 16S and once for *rpoD* and *gyrB*. NCBI nucleotide BLAST was used to search the good quality region of each sequencing read for matches.

### Phylogenetic trees

1414 bp of 16S rRNA, 740 bp of *rpoD* and 1028 bp of *gyrB* sequences of *P. stutzeri* single isolates PS4/5/8/13/14/15 and 69 *P. stutzeri* strains obtained from NCBI were concatenated, aligned and input into phylogenetic trees using MEGA (version 11.0.11), alignment was performed using the MUSCLE algorithm, distance was measured using pair‐wise *p*‐distance with standard settings, a neighbour‐joining tree was constructed using the *p*‐distance with standard settings. *Pseudomonas aeruginosa* PAO1 and *Pseudomonas putida* KT2440 were used as outgroups.

### Motility assays

Overnight cultures of *P. stutzeri* isolates were grown in LB at 30°C and OD corrected to OD_600_ 3, 5 μL was spotted on the surface of swarming assay plates (25 mL of LB with 0.5% agar, dried for 19 min next to a bunsen burner) and incubated upright at 30°C for 24 h. 0.5 μL of the OD_600_ 3 suspension was spotted into the middle of the agar of swimming assay plates (25 mL of tryptone 10 g/L, NaCl 5 g/L, agar 3 g/L, dried for 10 min next to a bunsen burner) and incubated upright at 30°C for 17 h. For twitching assays, single colonies from a streak plate grown overnight at 30°C were picked with a P200 pipette tip and stabbed to the bottom of the agar plate (10 mL of tryptone 10 g/L, yeast extract 5 g/L, NaCl 10 g/L, agar 10 g/L, dried for 8 min next to a bunsen burner) and incubated upright at 30°C for 48 h. Each biological replicate consisted of 3 technical replicates.

### Tetrazolium colorimetric assay

Overnight cultures grown in LB broth at 30°C were OD corrected to OD_600_ 0.5 and washed four times in 1 mL PBS with centrifugation for 3 min at 8000*g* between each wash. Pellets were resuspended in M9 minimal media with no carbon source supplemented with 0.2 mg/mL 2,3,5‐Triphenyltetrazolium chloride (Fisher) and 196 μL of each resuspension was added to 96‐well polystyrene plates containing 4 μL 20% glucose (positive control), 4 μL sterile water (negative control) or 4 μL sterile water and sterilized PCL bead (sterilized in 70% ethanol for 30 min), in triplicate. Plates were grown with agitation at 30°C for 7 days, and colorimetric changes observed.

### Growth with PCL and weight loss

Overnight cultures grown in LB broth at 30°C were OD corrected to OD_600_ 0.3 and washed three times in 1 mL PBS with centrifugation for 3 min at 8000 *g* between each wash. For the PCL growth curve, washed pellets were resuspended in 20 mL M9 minimal media with no carbon source and grown at 30°C with agitation for 4 weeks with and without PCL (13 beads, ~0.25 g, weighed prior to sterilization in 70% ethanol). For the PCL weight loss, at the end of the growth curve in M9 minimal media with no carbon source, the beads were collected, rinsed with distilled water, washed on a room temperature rocker in 2% sodium dodecyl sulphate overnight to remove biofilm, rinsed with distilled water and dried before weighing.

### Genome sequencing and analysis

The genome of PS13 isolate *P. stutzeri* was sequenced using Illumina NextSeq 2000 (SeqCenter, USA). Genome analysis and assembly for PS13 was completed in KBase using FastQC to assess read quality, SPAdes (v 3.15.3) to assemble the reads, followed by QUAST (v 5.2) and CheckM (v 1.0.18) to evaluate the resulting assemblies. Prokka (v 1.14.6) was employed for genome annotation, and genome mining to identify potential polyesterases was performed by command‐line BLASTP search (*e*‐value threshold of 1*e*−06) of the annotated protein output file against a custom database containing the amino acid sequences of known PET‐active enzymes (Camacho et al., 2008; Seemann, 2014). The genome mining strategy was executed similarly to as described previously (Carr et al., 2022), but here the 20 reference sequences used to construct the custom database (Table 1) were selected based on their inclusion in the Plastics‐Active Enzymes Database (PAZy) (entries with available PDB structures) (Buchholz et al., 2022). The mono(2‐hydroxyethyl) terephthalate hydrolase (MHETase) gene was identified by manual search of the keyword ‘terephthalate’ in the Prokka output, and the corresponding protein sequence was compared against the known MHETase from *Ideonella sakaiensis* (IsMHETase) using the 2‐sequences tool of the blastp online suite (Yoshida et al., 2021).

**TABLE 1:** Dataset of functionally verified PET‐active enzymes that was used to construct the custom database for genome mining.

Name	Source	GenBank/UniProt/MGnify
IsPETase	*Ideonella sakaiensis*	A0A0K8P6T7
PET6	*Vibrio gazogenes*	A0A1Z2SIQ1
Ple628	*Marinobacter* sp.	UUT36764.1
Ple629	*Marinobacter* sp.	UUT36763.1
PE‐H	*Pseudomonas aestusnigri*	A0A1H6AD45
PmC	*Pseudomonas mendocina*	N20M5AZM016
RgPETase	*Rhizobacter gumimpihilus*	A0A1W6L588
LCC	Leaf compost metagenome	G9BY57
BhrPETase	HR29 bacterium	GBD22443.1
TfH	*Thermobifida fusca*	Q6A0I4
Thc_Cut1	*Thermobifida cellulosilytica*	ADV92526.1
Thc_Cut2	*Thermobifida cellulosilytica*	ADV92527.1
Est119	*Thermobifida alba*	F7IX06
Cut190	*Saccharomonospora viridis*	W0TJ64
MtCut	*Marinactinospora thermotolerans*	WP_078759821.1
Enzyme 611	*Saccharopolyspora flava*	WP_093412886.1
BsEstB	*Bacillus subtilis*	ADH43200.1
PHL‐7	*Thermoanaerobacter* sp.	MBO2503201.1
PET30	*Kaistella jeonii*	WP_039353427.1
PET2	Metagenome‐derived (unassigned)	C3RYL0

Signal peptides were predicted for the candidate polyesterases using SignalP‐6.0 (Teufel et al., 2022) and removed prior to T‐COFFEE Expresso (Di Tommaso et al., 2011) structural amino acid sequence alignment of PsP1 and PsP2 with each of their top hits (PmC and MtCut, respectively). The alignment outputs were visualized in ESPript 3.0 (Di Tommaso et al., 2011), and the identification of active site features was facilitated by the Lipase Engineering Database (LED) PETase tool within the PAZy database. The key MHETase active site residues were identified based on a previous description of the substrate‐bound crystal structure (Palm et al., 2019). The SUPERFAMILY database was applied for protein classification (Pandurangan et al., 2019). Predicted protein structures were generated with Phyre2 (Kelley et al., 2015) and uploaded into PyMOL for visualization and alignment, using command cealign. Phyre2 predictions modelled 91%, 93% and 89% of residues at >90% confidence for PsP1, PsP2 and PsM1, respectively.

## RESULTS

### Biofilm formation on different plastic substrates

**FIGURE 1:**
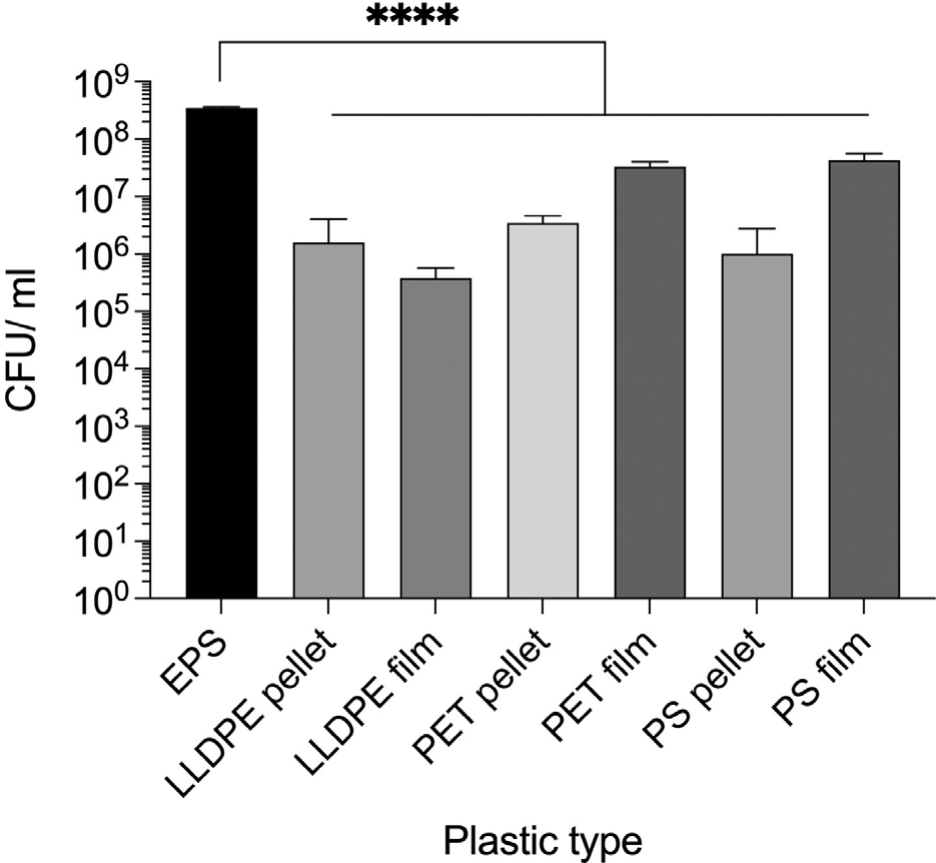
Bacterial biofilm formation on different plastic substrates. BL21 pCOLAduet‐1 *dgcC* formed significantly more biofilm on EPS than the other plastics tested. Mean and standard deviation (SD) from 3 pieces of plastic from individual cultures, one‐way ANOVA was performed with multiple comparisons, the difference between EPS and each of the other plastics was *p* < 0.0001.

We hypothesised that bacteria present on plastic pollution are more likely to possess plastic‐degrading abilities as the prevalence of plastic waste has previously been directly linked to prevalence of genes encoding putative plastic‐degrading enzymes (Zrimec et al., 2021). We aimed to identify the plastic type that promoted most bacterial biofilm formation, to increase the likelihood of identifying bacteria or communities with plastic‐degrading potential (Adoni et al., 2022; Artham et al., 2009; Atiq et al., 2010; Gilan et al., 2004; Lobelle & Cunliffe, 2011; Mor & Sivan, 2008; Morohoshi et al., 2018). Using pure untreated pellets and film of linear low‐density polyethylene (LLDPE), polyethylene terephthalate (PET) and PS as well as EPS, we tested bacterial attachment. We chose these plastics to represent common waste plastics of different structural and chemical types (Tsakona et al., 2021). We normalized the plastic by weight since plastic waste is normally reported in Mt. Additionally, measuring the surface area of the plastic can be inaccurate due to the variable surface topology at the nanoscale. To normalize the genetic background and ensure the capacity for equal levels of biofilm formation across the substrates, we used *E. coli* BL21 expressing a gene encoding the diguanylate cyclase *dgcC*, from an inducible plasmid. This encodes an enzyme that increases the intracellular levels of the second messenger signalling molecule, Cyclic‐di‐GMP (CdiGMP), which subsequently induces biofilm formation (Leech, 2017). After 24 h growth with 0.03 g of the plastic, EPS had significantly more bacterial biofilm formation than all other plastics, with an up to 3 log difference in numbers of recovered cells (Figure 1). A portion of this increase is likely to be due to the larger surface area of EPS compared to other plastics tested, however given that plastic waste is largely reported in Mt, this increased surface area is recapitulated in the natural environment also, whereby 1 Mt of EPS will have an increased surface area as compared to 1 Mt of PET. These high levels of bacterial biofilm formation suggest that EPS waste could be a good source for native plastic‐associated bacterial biofilm communities.

### Application of stringent selection pressures on native bacterial communities attached to coastal plastic waste leads to enhanced polyester‐degrading capacity

**FIGURE 2:**
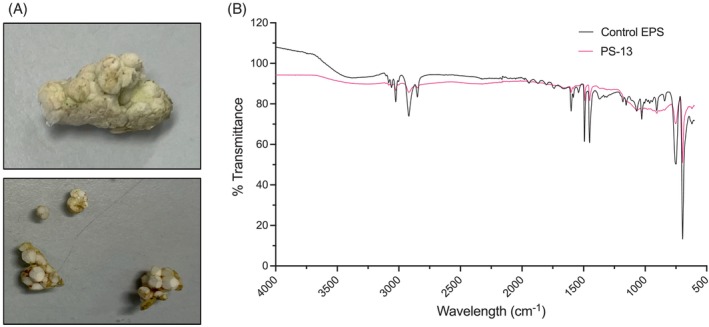
FTIR analysis of the plastic pollution samples confirms that they are polystyrene. (A) Examples of plastic pollution collected. (B) FTIR analysis of plastic waste PS‐13 (from which PS13 communities were collected) compared to pure EPS control. The EPS waste has PS characteristic peaks such as at 695, 754, 1451, 1492, 1601 and 2918 cm^−1^ (Fang et al., 2010; Peltzer & Simoneau, 2013).

Since EPS had the highest bacterial attachment per gram of waste plastic and therefore most bacteria associated with it, we sought out EPS waste as a rich source of plastic‐associated biofilms. Plastic pollution (16 pieces) washed up on the coast (51.8588° N, 8.0020° W) of the Republic of Ireland was collected. Specific pieces were selected based on visual similarity to EPS and FTIR was used to confirm this, the expanded polystyrene pieces were named PS1‐16. Using an automated NICODOM FTIR Spectra library, polystyrene was the closest match, confirming that the collected plastic waste is EPS (Figure 2).

**FIGURE 3:**
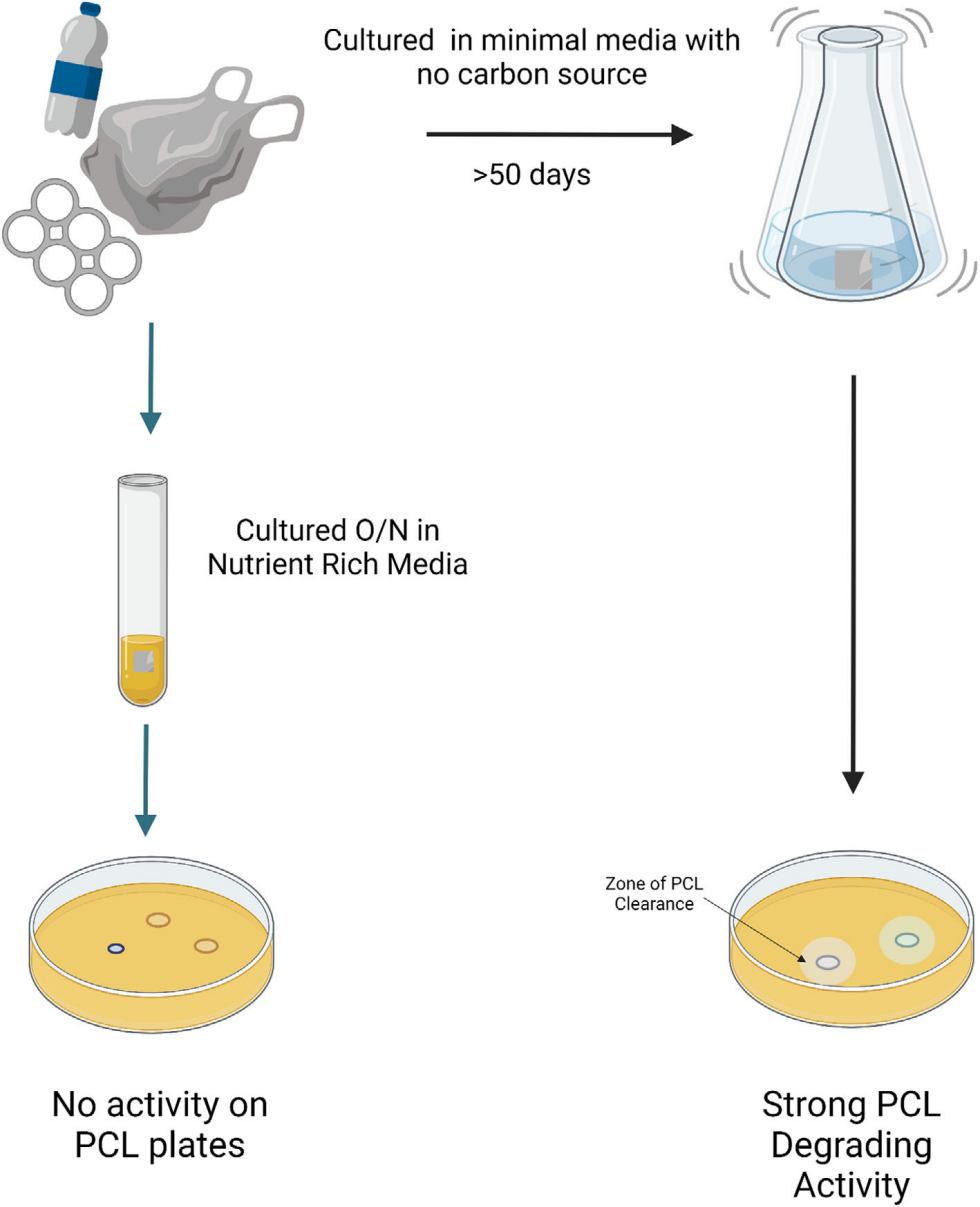
Experimental design. Waste plastic was collected from the coast and brought to the lab before being sectioned and added to nutrient rich media for growth overnight and cultures were then spotted onto PCL agar plates. Another section was put in high salt M9 minimal media with no carbon source and left to incubate for 53 days before plastic‐degrading activity was assessed.

**FIGURE 4:**
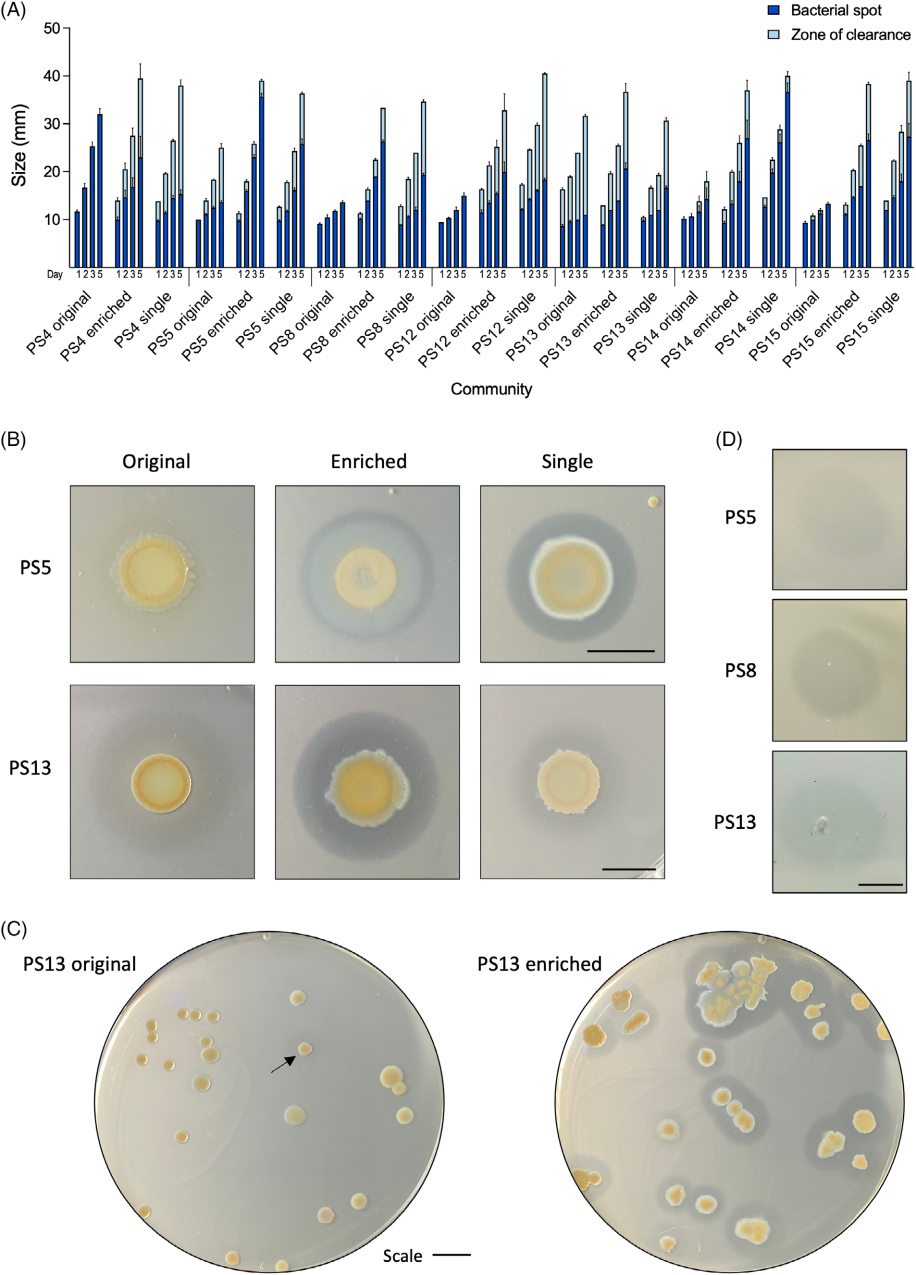
Bacteria enriched in polystyrene in minimal media have improved PCL degradation. Original communities are bacteria isolated from polystyrene samples after an overnight LB culture. Enriched communities are bacteria isolated from polystyrene samples after 53 days in high salt M9 minimal media with no carbon source, single isolates are the most efficient PCL‐degrading colonies from the enriched community. (A, B) 20 μL of OD_600_ 3 of an overnight LB culture of each strain was spotted onto PCL agar plates and grown for 5 days at 30°C, the bacterial spot size (dark blue) and PCL zone of clearance (light blue) was measured on days 1, 2, 3 and 5, mean and standard error of the mean (StEM) of 3 repeats. (B) A representative photograph of PS5 on day 2 and PS13 on day 3 to show clarity of the zone of clearance. (C) Colonies of PS13 original and PS13 enriched were plated on PCL plates to show proportion of colonies that could degrade PCL in the communities, imaged on day 3, arrow indicates faint zone of clearance for PS13 original. (D) The filtered supernatant of enriched PS5, PS8 and PS13 showed the ability to degrade PCL. All scale bars are 10 mm.

To assess the plastic degrading ability of the bacteria present in the attached biofilm communities, each piece of EPS was cut in half; one half was placed in a rich nutrient broth culture and grown overnight with agitation at room temperature, a sample of these cultures were then spotted on a PCL agar plate to screen for polyesterase activity through a zone clearance assay (Figure 3). PCL is a model substrate for the degradation of polyesters and can be used for high‐throughput screening purposes to assess the capacity of large numbers of bacteria to degrade polyester‐based plastic (Almeida et al., 2019; Danso et al., 2018; Nawaz et al., 2015). These cultures were also stocked in glycerol as the originally isolated community from the EPS waste. Of the 16 original communities, only EPS sample 5, 13 and 14 showed some slight PCL degradation, this was very faint and for PS14 only appeared after 3 days growth (Figure 4A–C). The other half of the EPS sample was placed in high salt M9 minimal media with no carbon source (NaCl concentration of 35 g/L, the same concentration as sea water) and grown for 53 days with agitation at room temperature. After 53 days, clear evidence of growth was visible in all the tubes. A sample of this culture was used to inoculate into rich nutrient broth and the culture grown overnight for glycerol stocks as the enriched community, and a sample spotted onto PCL agar plates to look for PCL degradation activity.

Seven of the 16 enriched communities showed a dramatic increase in PCL degradation activity (Figure 4A), suggesting that the native communities had been enriched to have increased plastic‐degrading potential over the course of the experiment. These enriched communities were streaked for single colonies on PCL agar plates to identify the specific bacteria that produced the largest zone of PCL clearing. A single colony was selected from each enriched community that showed good PCL degradation and had similar zones of clearance to the enriched community, and in some cases were better, such as for PS4, PS5, PS8 and PS12 (Figure 4A–C), suggesting antagonism may be taking place within the enriched communities that was relieved when strains were grown in isolation.

To gain a better understanding of this observation at a single colony level rather than community level, one of the best performing samples, PS13 was selected for further analysis. Approximately 150 colonies of the original and enriched communities of PS13 were compared to determine what percentage of each community could degrade PCL individually. After 2 days, no single colonies of PS13 original community could degrade PCL, this increased to 4.8% of colonies after 3 days, however these zones were very faint (Figure 4C). Conversely, after 2 days, 51.2% of single colonies from PS13 enriched communities were able to degrade PCL, increasing to 94.7% after 3 days, for which all zones were very clear. The evidence that after 2 days no bacteria displayed PCL‐degrading activity in the original community, suggests that bacteria with polyester degrading ability are very low in numbers or that the polyester‐degrading enzymes are only weakly expressed.

To determine if the enzymes associated with this PCL degrading activity were secreted and could therefore contribute to a community level synergistic plastic catabolizing pathway, the positive degrading enriched communities were tested for supernatant activity on PCL. Supernatant from overnight cultures of PS5, PS8 and PS13 displayed supernatant degradation of PCL (Figure 4D), showing that these cultures most likely secrete enzymes responsible for PCL degradation.

### Identification of native biofilm plastic‐associated communities

**FIGURE 5:**
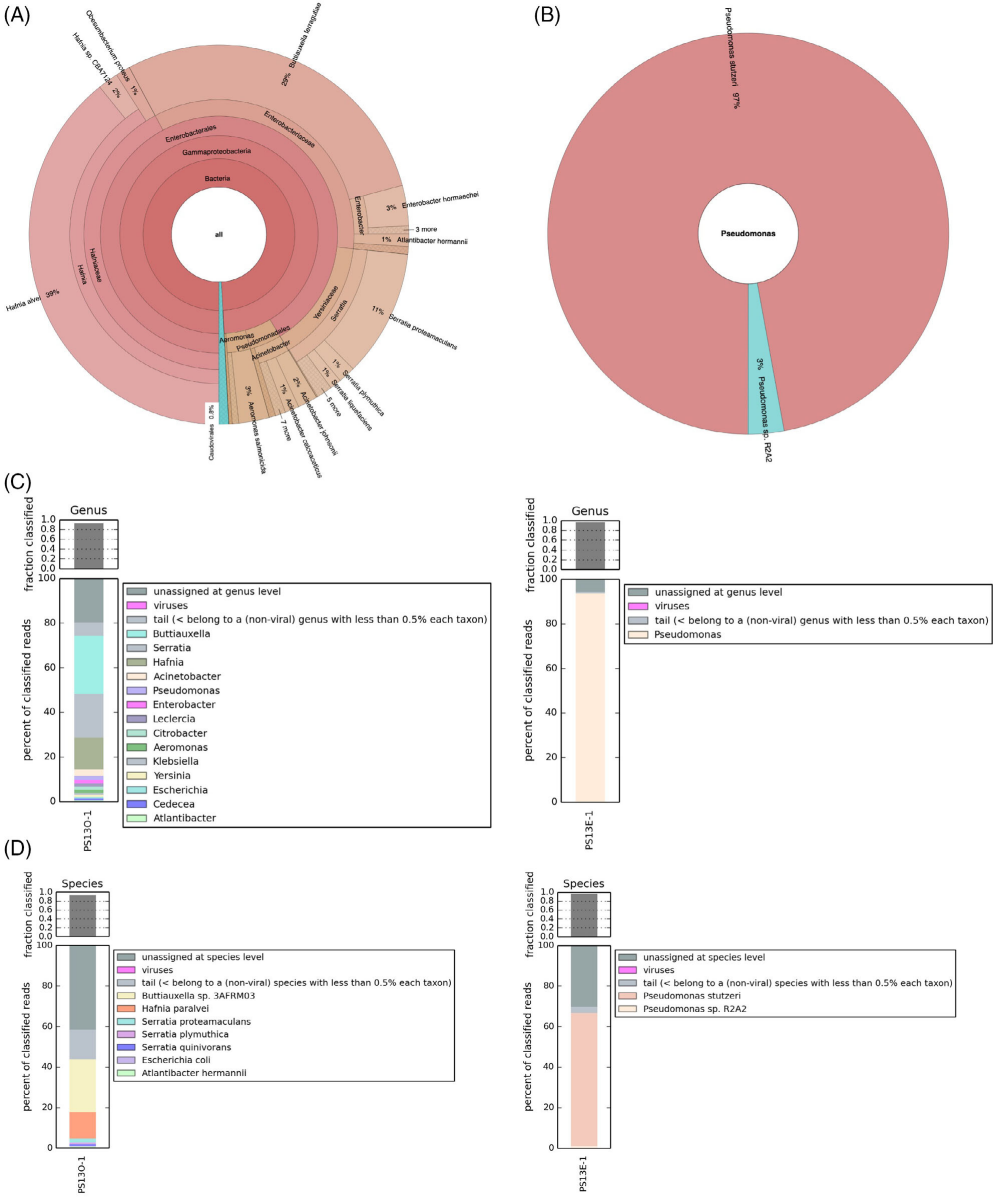
Community analysis of PS13 original and enriched. Krona chart displaying the relative abundance and diversity at the species level of (A) the PS13 original dataset, and (B) the PS13 enriched dataset, generated by GOTTCHA2 from Illumina sequence reads. Kaiju‐generated stacked barplots representing taxonomic classification of PS13 original (PS13O) (shown on left) and PS13 enriched (PS13E) (shown on right) at (C) genus level and (D) species level.

The PS13 community was chosen for further study due to its large zone of clearance. Community analysis was performed for the originally isolated community and the enriched community of PS13. The taxonomy of the PS13 original and PS13 enriched communities were visualized in Krona charts generated using GOTTCHA2 (Figure 5A,B). There was a clear difference observed between the PS13 original (Figure 5A) and PS13 enriched (Figure 5B) samples, whereby the original community features a range of members from *Enterobacteriaceae* (including *Buttiaxella*, *Enterobacter* and *Klebsiella* genera), *Hafniaceae* (predominantly *Hafnia* genera), and *Yersiniaceae* (predominantly *Serratia* genera) families, while the enriched community appears much less diverse, primarily featuring members of the genus *Pseudomonas*. Within the PS13 original community, several different species are found in high relative abundances, including *Hafnia alvei* (39%), *Buttiaxella ferragutiae* (29%), *Serratia proteamaculans* (11%), *Aeromonas salmonicida* (3%) and *Acinetobacter johnsonii* (2%). By comparison, the data for PS13 enriched reveals a very high relative abundance of *Pseudomonas stutzeri* (97%), along with a smaller proportion of *Pseudomonas* sp. R2A2 (3%). The stacked barplots for genus (Figure 5C) and species (Figure 5D), which were generated using Kaiju, portray a similar overall trend such that diversity has decreased following the enrichment experiment. In both graphical representations, the community has undergone notable change in composition with *P. stutzeri* emerging as the dominant species in the PS13 enriched sample.

### Identification of species with capacity to degrade plastic

**FIGURE 6:**
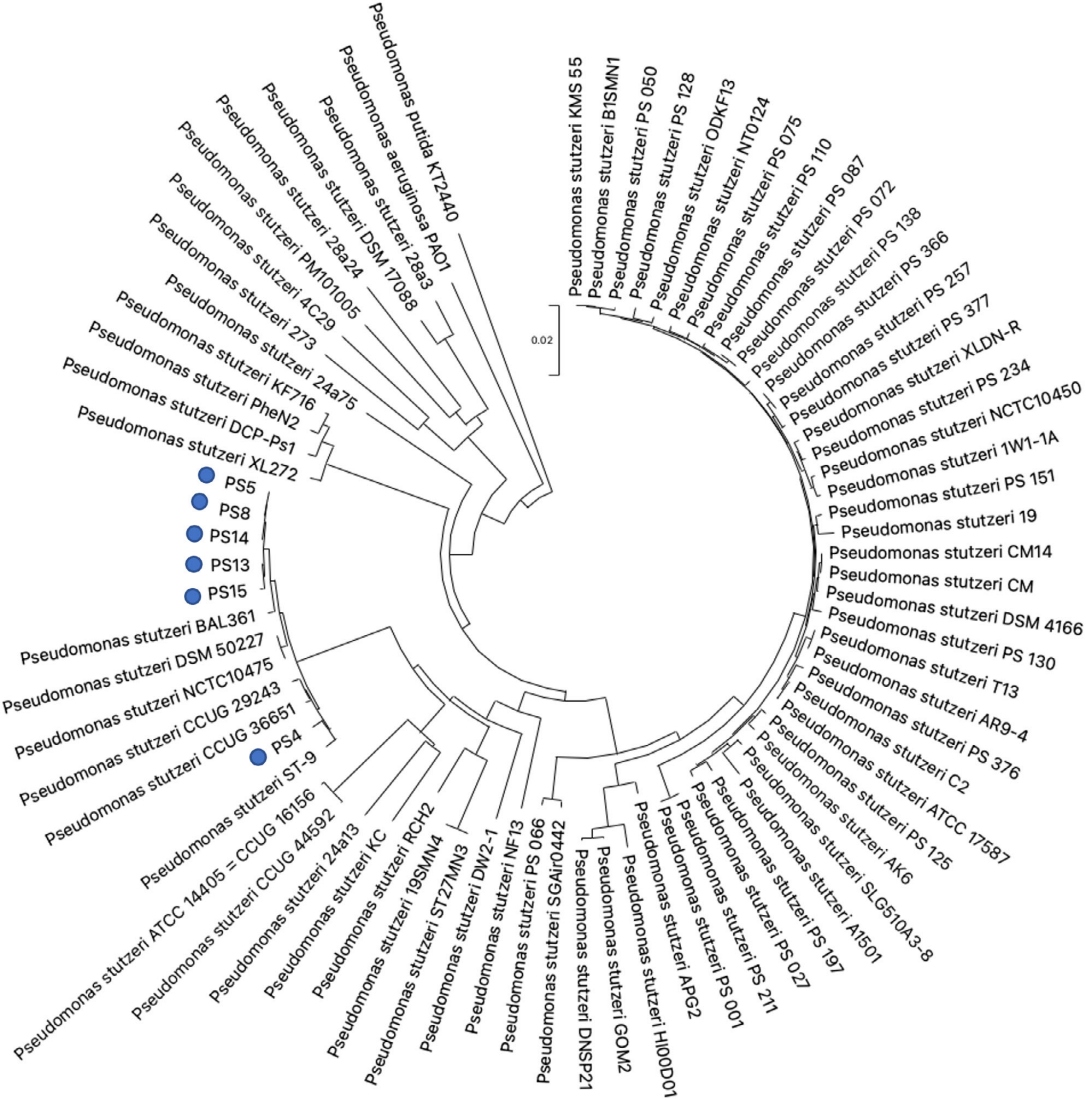
Phylogenetic tree of *Pseudomonas stutzeri* isolates obtained from enriched communities. A neighbour‐joining tree was constructed using the *p*‐distance of concatenated 16s rRNA, *rpoD* and *gyrB* sequences of PS4/5/8/13/14/15 and 69 *P. stutzeri* strains. *Pseudomonas aeruginosa* PAO1 and *Pseudomonas putida* KT2440 were used as outgroups. Enriched plastic degrading isolates highlighted with blue dots.

As the enriched PS13 community is predominantly *P. stutzeri*, we performed 16S rRNA, *gyrB* and *rpoD* sequencing of the previously identified best PCL degraders from the seven enriched communities to identify the other species that are able to degrade PCL. Remarkably, this sequencing revealed that the dominant PCL degrading organism in six of the seven enriched communities was also *P. stutzeri*. This was an unexpected finding as *P. stutzeri* has not been characterized as being able to degrade petroleum‐based polyesters previously, and as a potential organism for plastic bioremediation is relatively understudied compared to other members of the *Pseudomonas* genus such as *Pseudomonas fluorescens* (Howard & Blake, 1998; Hussain et al., 2015). The sequencing showed that the isolates (PS4/5/8/13/14/15) are closely related and cluster together when phylogenetic analysis was performed on the concatenated sequences (Figure 6). Even though these isolates have highly similar sequences, they have differing efficiencies at degrading PCL (Figure 4A) and are morphologically distinct (Figure S1). Motility assays also show further phenotypic differences between the strains (Figure S2). Differences are expected as the isolates were from different plastic‐associated biofilms, but it is interesting that in six enrichment experiments, *P. stutzeri* became the dominant species. Only the PS12 single isolate was a different genus, identified as 100% match to many strains of *Bacillus* species. Bacillus have been quite well characterized as plastic‐degrading bacteria (Arkatkar et al., 2010; Mohan et al., 2016; Ribitsch et al., 2011; Rowe & Howard, 2002; Yang et al., 2014).

### *P. stutzeri* can use PCL as its sole carbon source

**FIGURE 7:**
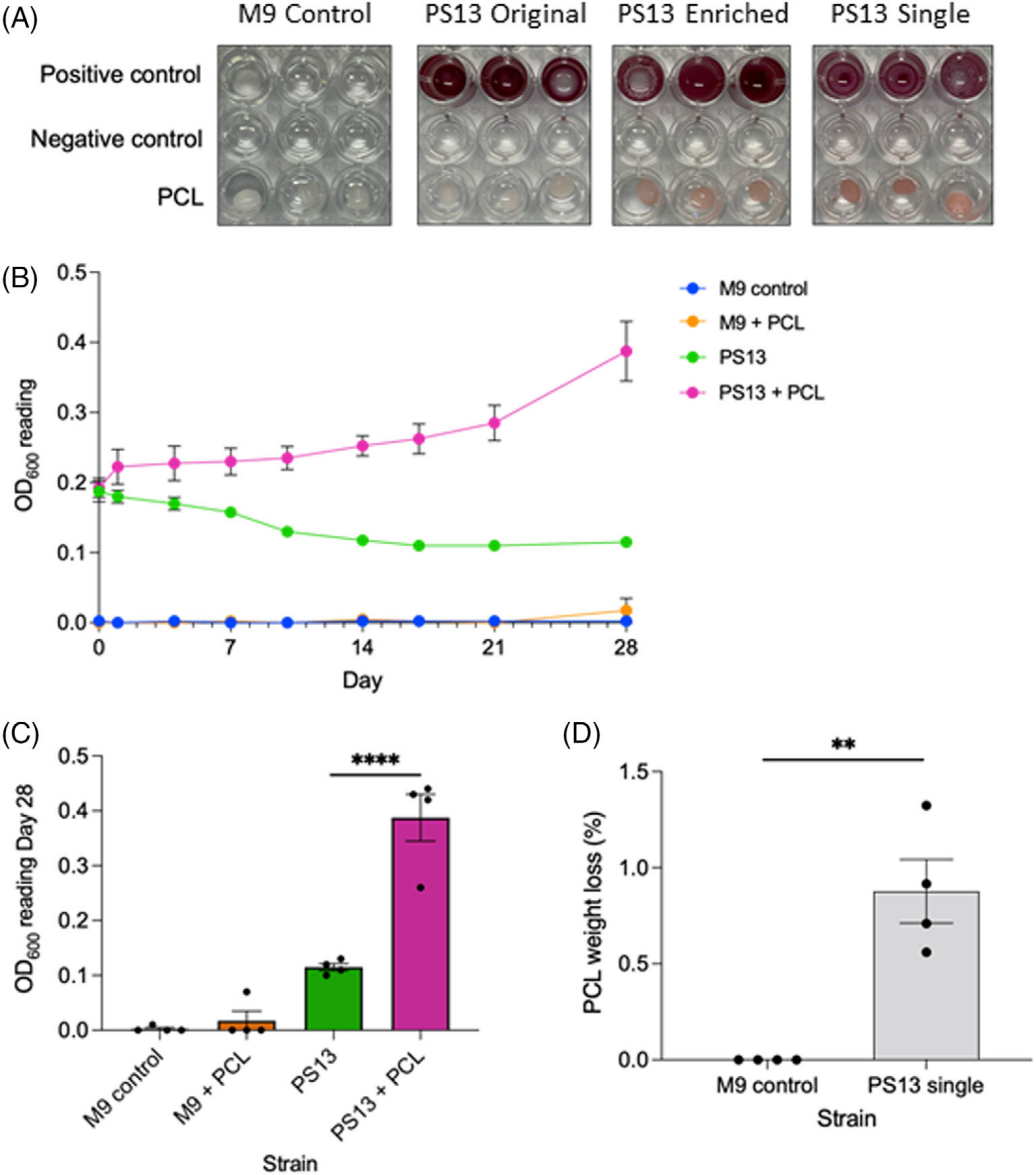
*Pseudomonas stutzeri* PS13 uses PCL as a carbon source. (A) Metabolic assay in M9 minimal media containing 0.2 mg/mL 2,3,5‐triphenyltetrazolium chloride to show use of PCL bead as a sole carbon source. Positive controls have 0.4% glucose, negative controls have no carbon source. Representative of six independent repeats for original and enriched communities, three for single isolate, imaged after 7 days growth at 30°C. The PS13 enriched community and single isolate are able to metabolize PCL well, shown by the pink colouring of the PCL surface, whereas the PCL bead is only slightly coloured pink by the original PS13 community. (B) Growth curve of PS13 single isolate *P. stutzeri*, mean and StEM of four independent repeats in M9 minimal media (no carbon source) with and without PCL. (C) Statistical comparison of growth curve day 28, two‐way ANOVA with Tukey multiple comparisons performed, *p*‐value <0.0001. (D) Weight loss of PCL from four 28‐day growths with PS13 *P. stutzeri*, unpaired *t*‐test, *p*‐value 0.0018.

Enriched PS13 single isolate *P. stutzeri* was chosen for further study due to its high zone of clearance on PCL and supernatant degradation of PCL. A metabolic colorimetric assay using 2,3,5‐Triphenyltetrazolium chloride to detect metabolically active cells was used to show that the enriched PS13 community and single isolate *P. stutzeri* are able to metabolize PCL, shown by the pink colouring of the PCL bead when provided as the sole carbon source (Figure 7A). The originally isolated community shows only a slight colouration of the bead, which corresponds with the minor levels PCL degradation present with this community (Figure 4A–C). It is interesting that the colouration is specifically on the bead surface indicating that the bacteria was attached to and forming a biofilm on the surface of the PCL bead.

Growth curves of PS13 single isolate *P. stutzeri* in M9 minimal media (no carbon source) with and without PCL showed that this isolate is able to grow using PCL as a sole carbon source (Figure 7B). From four independent 28‐day growth experiments, *P. stutzeri* PS13 always increases in OD_600_ when given PCL as the sole carbon source compared to the control where no carbon source is present and the OD_600_ decreases. End point analysis at 28 days showed that growth of PS13 single isolate was significantly higher in cultures with PCL as compared to those without (Figure 7C). PCL was collected after 28 days incubation with PS13 *P. stutzeri* in M9 minimal media with no carbon source and was washed and weighed, showing the average and StEM weight loss of PCL was 0.877 ± 0.166% (Figure 7D).

Given that we sought out EPS waste specifically based on the high levels of bacterial biofilm formation on this substrate, we wanted to confirm whether *P. stutzeri* also attached more to EPS than other plastic types. The same attachment assay was performed, as with *E. coli*, *P. stutzeri* also attached most to EPS compared to the other plastic types tested (Figure S3). We also assayed EPS weight loss with the PS13 enriched community and a general trend of weight loss was observed; however, there was significant variation between replicates (Figure S4).

### Whole‐genome analysis of *P. stutzeri* PS13

To further characterize *P. stutzeri* PS13, we performed whole‐genome sequencing with the aim to search for potential plastic‐degrading enzymes. The overall quality of the raw genome sequence data was evaluated using FastQC prior to assembly and annotation, and a high‐quality draft genome was subsequently constructed in 12 contigs for the PS13 strain, with 100% completeness and 0.14% contamination (Table S1). The genome properties, such as the size (4.61 Mb) and the GC content (62.92%), are similar to that observed for other *P. stutzeri* strains (Chakraborty et al., 2017; Li et al., 2022). A total of 4291 coding sequences were annotated by Prokka, including 1721 hypothetical proteins.

Two genes encoding potential polyesterase homologues were identified during genome mining of PS13, which we named ‘*Pseudomonas stutzeri* polyesterase 1 and 2’ (PsP1‐2) (Table 2). PsP1 produced significant alignments with 16 of the 20 enzymes in the custom PET‐active database, where the top hits were PmC, Est119, PE‐H and LCC, while for PsP2, significant alignments were detected against two of the reference sequences, MtCut and Est119. Additionally, a putative MHETase was identified in the PS13 genome, and designated as ‘PsM1’.

**TABLE 2:** Polyesterase homology search output generated by BLASTP of the Prokka‐annotated proteins found in the PS13 genome.

Prokka locus tag	Enzyme name	Annotation	Length	Top hits	Score	*e* value	Identity (%)
ALPBGIHC_00532	PsP1	Hypothetical protein	282	PmC	426	9*e*−156	82
				Est119	62.0	2*e*−14	29
				PE‐H	61.6	4*e*−14	29
				LCC	60.8	6*e*−14	29
ALPBGIHC_02632	PsP2	Hypothetical protein	295	MtCut	49.3	5*e*−10	28
				Est119	41.6	2*e*−07	27

**FIGURE 8:**
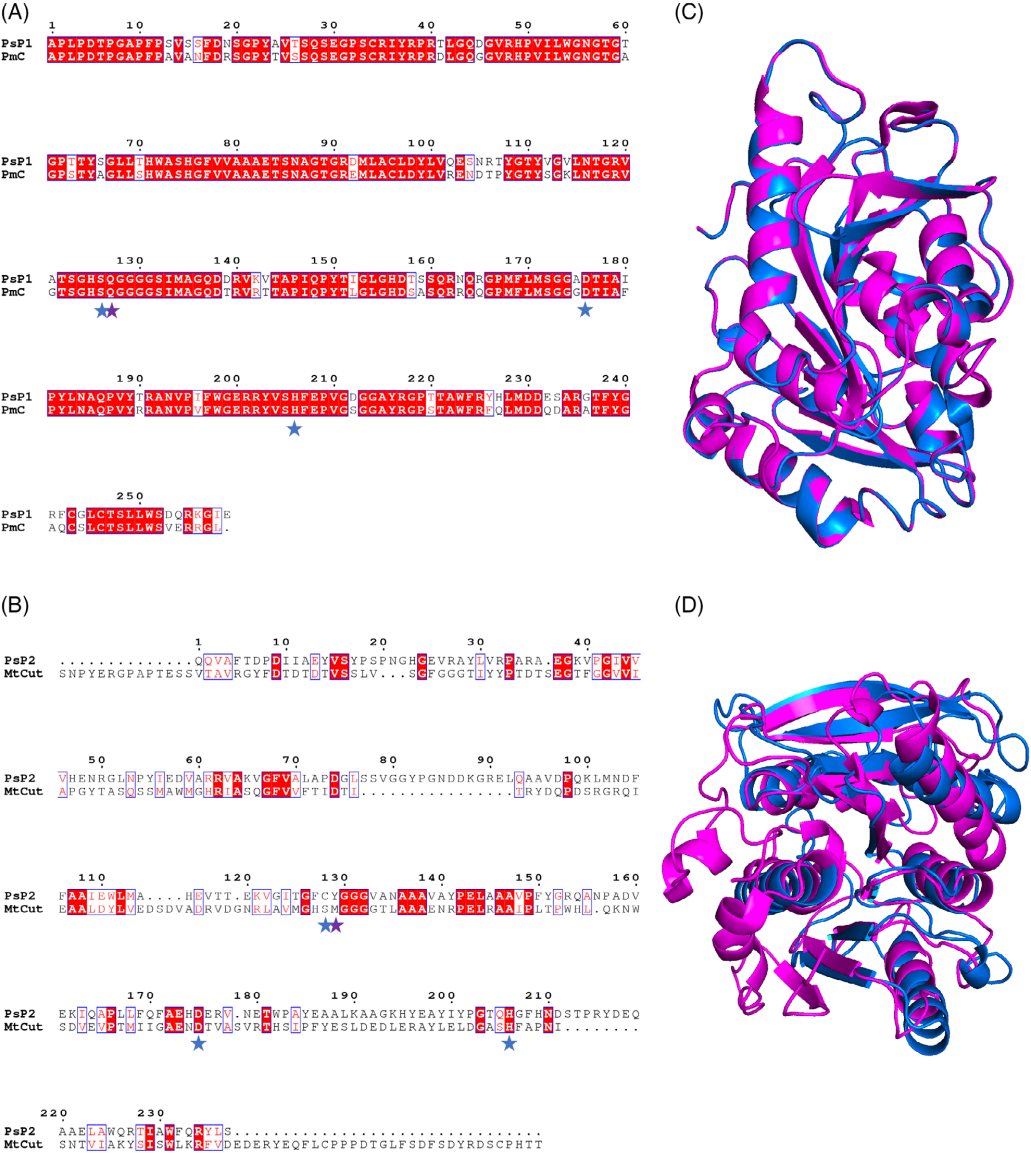
Protein sequence structural alignment of *P. stutzeri* PS13 putative polyesterases. (A) PsP1 and PmC, and (B) PsP2 and MtCut, generated using T‐COFFEE in Expresso mode and rendered in ESPript 3.0. Amino acid residues shaded in red represent the ones strictly conserved between sequences, while those outlined in blue share average levels of homology. Catalytic triad residues are marked with blue stars, while oxyanion hole residues are marked by purple stars. (C) PyMOL structural alignment of Phyre2 predicted PsP1 (blue) and PmC (PDB 2FX5) (magenta) with a RMSD of 0.140436 over 256 residues. (D) PyMOL structural alignment of Phyre2 predicted PsP2 (blue) and MtCut (PDB 7QJO) (magenta) with a RMSD of 3.920728 over 200 residues. Predicted signal peptides have been removed from alignments.

Both PsP1 and PsP2 were classified as members of the α/β hydrolase superfamily, which has generally been observed for the polyesterases reported to date (Gricajeva et al., 2022). Based on the T‐COFFEE structural alignment of PsP1 against PmC, it was inferred that 81.47% of residues were identical between the two sequences, while 93.44% of residues shared similar biochemical properties (Figure 8A). The Ser‐Asp‐His catalytic triad and an oxyanion hole formed by Gln are among the residues conserved between PsP1 and PmC. For PsP2 and MtCut, the sequences were found to share a mean identity of 14.83%, with a mean similarity of 64.14% (Figure 8B). While MtCut features the typical Ser‐Asp‐His catalytic triad, PsP2 possess a nucleophilic cysteine in place of serine to give a Cys‐Asp‐His catalytic triad. The adjacent oxyanion hole also differs between the two sequences, formed by Met in MtCut and Tyr in PsP2. Structural alignment of PsP1 and PmC in PyMOL gives a root mean square deviation (RMSD) of 0.140436, further showing that they are highly structurally similar (Figure 8C). The RMSD of PsP2 with MtCut is 3.920728, reflecting the lower sequence homology (Figure 8D).

**FIGURE 9:**
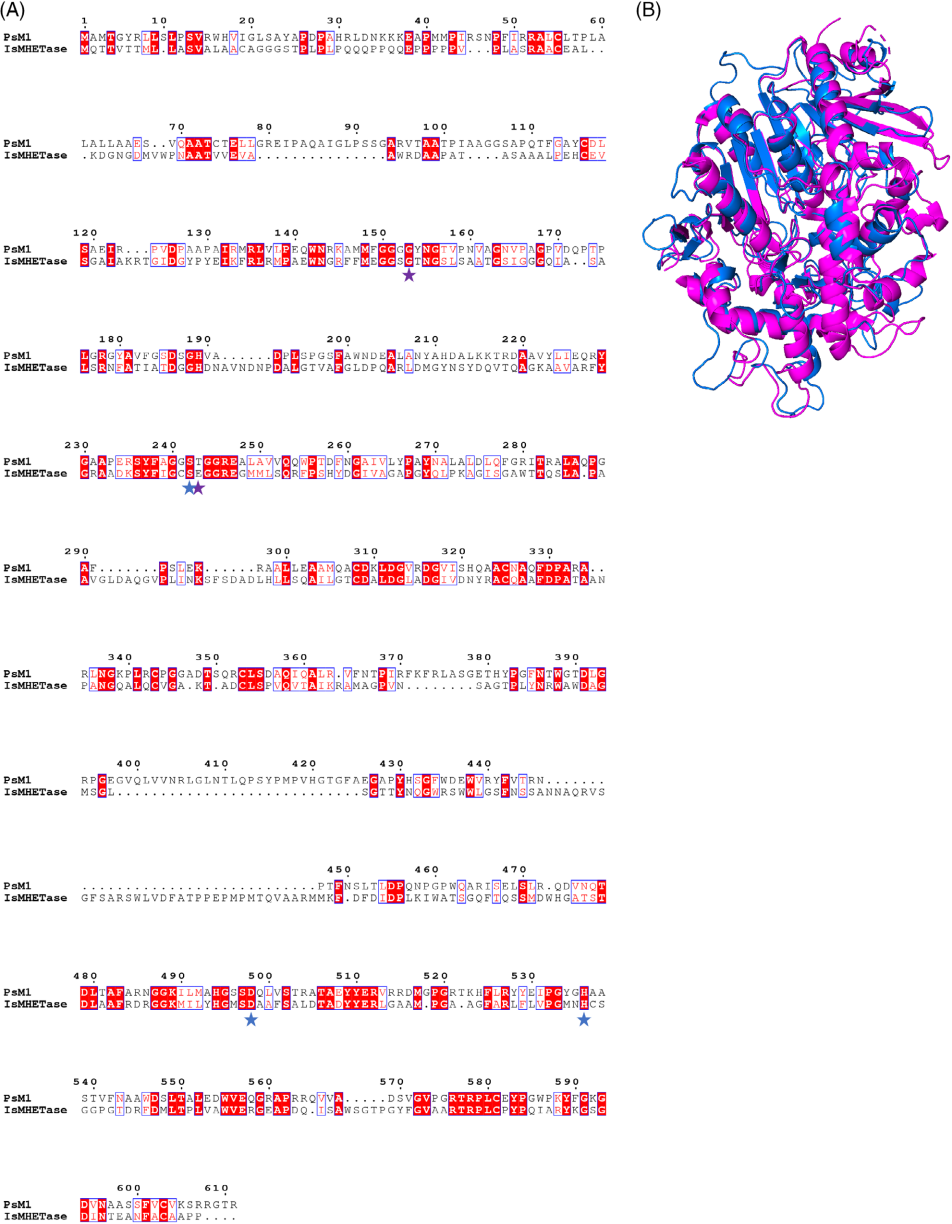
Protein sequence structural alignment of PsM1 and IsMHETase. (A) Generated using T‐COFFEE in Expresso mode and rendered in ESPript 3.0. Amino acids shaded in red represent identical residues, while those outlined in blue share similar biochemical properties. Catalytic triad residues are marked with blue stars and oxyanion hole residues are marked by purple stars. (B) PyMOL structural alignment of Phyre2 predicted PsM1 (blue) and IsMHETase (PDB 6QGB) (magenta) with a RMSD of 3.631573 over 504 residues, it is noted that IsMHETase structure starts on residue 43, which may increase the RMSD value.

The MHETase‐like enzyme, PsM1, was also classified as an α/β hydrolase superfamily member and its structural alignment with IsMHETase revealed 25.66% identity and 69.84% similarity (Figure 9). Here, the Ser‐Asp‐His catalytic triad and an oxyanion‐forming Gly residue are shared by PsM1 and IsMHETase, while the Glu oxyanion hole residue in IsMHETase is replaced by Thr in PsM1. The RMSD of PsM1 with IsMHETase is 3.631573, reflecting the lower sequence homology.

## DISCUSSION

Plastic pollution is a major threat facing global ecosystems. Novel solutions to tackle this problem are urgently needed as current recycling strategies cannot cope with the volumes of plastic entering waste streams. Bacterial bioremediation is an innovative approach that may become part of the solution to the plastic waste crisis. However, many of the bacterial strains that have been identified with this plastic‐degrading activity do so at very low rates over prolonged periods of time. Here, we found that bacterial biofilm formation was highest per gram on EPS and that EPS waste is a good source of natural plastic‐associated bacteria. We found that these bacteria also have the ability to degrade polyester. Waste marine plastic is known for being quite heavily colonized with biofilm, and biofilms can develop quickly in marine environments (Basili et al., 2020; Lobelle & Cunliffe, 2011). EPS may be a good substrate for bacterial attachment because of its many air pockets and uneven surface, providing protected spaces for bacteria. Additionally, EPS floats and will therefore be at the air‐water interface in the ocean, allowing growth of aerobes. It also travels long distances as it is lightweight (Schwarz et al., 2019). These factors would expose more diverse bacteria to this habitat, creating a highly diverse EPS plastisphere. Presence at the air‐water interface would also expose the EPS to more photo‐oxidation, which may promote degradation of the plastic and aid bacterial growth (Ward et al., 2019; Yousif & Haddad, 2013). EPS is also a highly prevalent plastic pollutant in the oceans (Chitaka & von Blottnitz, 2019; Esiukova, 2017; Hinojosa & Thiel, 2009; Moore et al., 2001; Tsakona et al., 2021; Zhou et al., 2020). As prevalence of plastic waste is directly linked to prevalence of plastic‐degrading genes, it is reasonable to hypothesise that bacteria colonizing plastic waste are also more likely to possess plastic‐degrading genes (Zrimec et al., 2021). Zrimec et al. (2021) showed that more genes with plastic‐degrading potential were present in areas with more plastic pollution, however, function and activity was not confirmed in their study. Some enzymes may be poorly expressed, only expressed under certain conditions or could be similar to confirmed enzymes but non‐functional. This could be where the enrichment experiment tested in our study could be used to unlock these enzymes, not only selecting for bacteria that possess them but potentially providing the selection pressure to change expression patterns or select for mutations in the genes that make them functional or drive the development of community level synergistic plastic catabolizing pathways.

Here, we show that laboratory‐based enrichment of environmental bacterial communities can augment their capacity to degrade plastic by applying a strong selective pressure such as providing plastic as a sole carbon source. This is important as even if native bacterial communities do not initially show signs of biodegradation, they could have the potential for biodegradation due to their prior exposure to plastic waste. We found that *P. stutzeri* was the dominant strain after the enrichment experiment, suggesting this species could be a natural environmental source for plastic degradation activity. Community enrichment in the laboratory where the carbon source is restricted to only plastic provides a novel method for improving biodegradation of environmental bacteria to levels that could be sufficient for use in bioreactors, improving on nature. This is crucial step in progressing bacterial biodegradation into practical industrial applications.

*Pseudomonas* species are well documented to degrade different plastics (Wilkes & Aristilde, 2017). Polyurethanases have been found in *Pseudomonas chlororaphis*, *P. aeruginosa*, *P. fluorescens* and *Pseudomonas protegens* which allow the bacteria to grow on polyurethane and degrade it (Howard & Blake, 1998; Hung et al., 2016; Ruiz et al., 1999; Ruiz & Howard, 1999; Shah et al., 2013). Furthermore, *Pseudomonas* sp. AKS2 has been shown to degrade low density polyethylene (LDPE) by 5% and a *Pseudomonas* spp. was able to degrade high impact polystyrene (HIPS) by <10% (Mohan et al., 2016; Tribedi & Sil, 2013). *P. aeruginosa* has also been shown to degrade polyethylene and the enzyme PE‐H from *P. aestusnigri* degrades PET (Bollinger et al., 2020; Mouafo Tamnou et al., 2021). *P. stutzeri* has previously been studied for biodegradation properties, however it has not been specifically linked with petroleum‐based polyester degradation. *P. stutzeri* has been found to be able to degrade polyhydroxybutyrate, a bio‐derived plastic (Kasuya et al., 1999). Veethahavya et al. (2016) studied *P. stutzeri* as part of a consortia of a 10 species of bacteria, the consortia were shown to degrade LDPE, but specific contributions of the individual consortia strains were not assessed. In another study, *P. stutzeri*, which was identified through morphological and biochemical properties rather than sequencing, was shown to grow in the presence of polypropylene (PP) and LDPE but no degradation of the plastic was measured, only reduction in tensile strength, percent extension and elongation of the plastic (Sharma & Sharma, 2004). Conversely, Arkatkar et al. (2010) studied the capability of *P. stutzeri* to degrade PP but found that it was unable to grow in the presence of this polymer or degrade it. This could be due to strain specific differences, especially if certain environmental strains have been exposed to plastic pollution and developed the ability to degrade it and others have not.

The majority of studies on native plastic‐associated biofilms do not experimentally assess plastic degradation abilities of these communities (Basili et al., 2020; Kirstein et al., 2019; Lobelle & Cunliffe, 2011; Mohsen et al., 2022; Pinto et al., 2019; Wallbank et al., 2022; Zettler et al., 2013). A few studies have characterized polystyrene‐associated bacterial communities. Syranidou et al. (2017) and Ogonowski et al. (2018) found that proteobacteria were the dominant phylum, as with our dataset, however, within this the majority of the PS13 original community was Gammaproteobacteria, whereas the other studies had more diverse classes. Kesy et al. (2016) found that the dominant species on PS buried in sediment were *Desulfatibacillum alkenivorans*, *Synechococcus* sp. and *Amphritea atlantica*. Another study that focused on culturable species on PS identified *Pseudomonas*, *Pseudoalteromonas*, *Halomonas* and *Alteromonas* (Laganà et al., 2019). Of the genus that we found in our PS13 originally collected sample, *Serratia*, *Hafnia* and *Klebsiella* have all been linked with PS previously. In yellow mealworms and superworms that were fed a PS diet, *Hafnia* and *Klebsiella* became the dominant genera, respectively, showing a strong link between PS and these genera (Wang et al., 2022). *Serratia* species has previously been identified in the gut flora of PS‐fed beetle larvae and found to cause cavities in PS film (Woo et al., 2020). Additionally, on the species level, the original PS13 community consisted of 2% *A. johnsonii*, interestingly, this species has previously been found to grow using PS and to degrade it (Kim et al., 2021). *P. stutzeri* was not identified in the original community, the community had been grown in LB media twice before DNA extraction (to collect it from the EPS sample and to grow it for DNA extraction), therefore it is likely that *P. stutzeri* may have been outcompeted within this time frame.

Many studies that identify plastic‐degrading bacteria have isolated them from the environment, however these are usually from soil or water from plastic contaminated areas rather than the plastic directly (Balasubramanian et al., 2010; Dey et al., 2020; Jeon & Kim, 2016; Kumari et al., 2019; Latorre et al., 2012; Nair & Kumar, 2007; Skariyachan et al., 2014; Skariyachan et al., 2018; Tribedi et al., 2012; Usha et al., 2011; Yoshida et al., 2016). A few studies have isolated plastic‐degrading bacteria directly from plastic; however, these introduce a controlled plastic to the environment for a set time rather than collect environmental waste plastic (Artham et al., 2009; Atiq et al., 2010; Gilan et al., 2004; Morohoshi et al., 2018). Here, we isolated the bacteria directly from biofilms associated with environmental waste plastic and showed that selection pressures could be applied to enhance polyesterase activity. By using native plastic‐associated communities to isolate plastic‐degrading bacteria, it should not only select bacteria that have a higher chance of encoding enzymes with the potential to degrade plastic (Zrimec et al., 2021), but also those that are better able to form biofilm on plastic, an important step for biodegradation (Flemming, 1998; Gilan et al., 2004; Han et al., 2020; Mor & Sivan, 2008; Morohoshi et al., 2018; Sivan et al., 2006).

We did test EPS degradation and while a trend was observed, there was a high level of variability between replicates under our conditions. The PCL degradation observed would not necessarily translate into PS degradation as they are different types of plastics. PCL is a biodegradable aliphatic polyester that is degraded through breakage of ester bonds, which is also the mechanism of action of PET‐hydrolysing enzymes (Albertsson & Varma, 2002; Liu et al., 2019; Sulaiman et al., 2012). PS on the other hand, which is made from styrene, an aromatic hydrocarbon, has a C–C backbone and unknown enzymes are responsible for previously reported biodegradation (Ho et al., 2018; Hou & Majumder, 2021; Ward et al., 2006). Polystyrene is particularly resistant to biodegradation and often has better biodegradability when mixed with starch or prooxidants (Ho et al., 2018; Jasso G et al., 1998; Kim et al., 2021; Ojeda et al., 2009; Schlemmer et al., 2009). The variable levels of EPS degradation could be because the waste EPS collected from the environment had been exposed to UV and potentially many chemicals and compounds in the environment that could have partially degraded it, making it easier for the bacteria to further degrade, whereas the EPS used in the laboratory is pure and undegraded (Lee et al., 2022; Song et al., 2020). In future studies, we could test different EPS, such as those that have been pre‐treated with UV, using weathered EPS waste, or using EPS mixed with starch or prooxidants, to assess if the bacteria are then able to degrade it. An additional factor that could influence the ability to degrade EPS is synergy between different species, other bacteria that may not have grown in our experimental set‐up may be required to work with our enriched communities to degrade EPS. Different species of bacteria have been shown to work together to degrade plastic previously (Aravinthan et al., 2016; Joshi et al., 2022; Roberts et al., 2020; Skariyachan et al., 2014; Syranidou et al., 2017; Veethahavya et al., 2016). The degradation process may also be very slow and not detectable in these experimental conditions and while the bacteria may be able to degrade polystyrene sufficiently to survive for 2 months, it may not result in detectable weight loss. Therefore, future experiments will focus on degradation studies over longer period of times (e.g. >6 months). An additional limitation to our study is that there is the possibility that the stringent selection pressure of the experiment induced community enrichment and survival regardless of EPS presence. This could be explored in future studies to assess how communities adapt and enrich in different conditions or on different plastic.

Interestingly, whole genome sequencing of *P. stutzeri* PS13 identified two putative polyesterases and a putative MHETase which could be responsible for the polyesterase activity seen for this strain. It is relatively unusual to identify a species of bacteria that encodes all the enzymes required for the full catabolism of PET and to the best of our knowledge neither a polyesterase or an MHETase has been described in *P. stutzeri* previously. The putative polyesterases PsP1 and PsP2, share most homology to known polyesterases PmC and MtCut, respectively. While lipase and esterase‐type polyesterases have been found to degrade petroleum‐based plastics, such as PET, the most efficient PET‐active enzymes are generally reported as cutinases or cutinase‐like enzymes (Kawai et al., 2020). *P. mendocina* cutinase (PmC) was studied alongside fungal cutinases HiC and FsC to assess catalytic activity towards PET films, and of these enzymes, PmC was found to have the highest substrate affinity (Ronkvist et al., 2009). More recently, MtCut, derived from a deep sea *Marinactinospora thermotolerans* strain, was reported as a novel cutinase that efficiently hydrolyses PET at ambient temperatures (Liu et al., 2022). With regards to enzymatic PET hydrolysis, MHET tends to emerge as the primary reaction product, and its accumulation can limit the full degradation of PET to its simplest monomers (Schubert et al., 2023). Thus, strains like *I. sakaiensis* which possess both a PET‐degrading enzyme and a secondary MHET‐degrading enzyme, are of interest for the complete hydrolysis of PET plastic (Palm et al., 2019). The appearance of two polyesterase homologues, as well as a MHETase‐like enzyme in the genome of *P. stutzeri* (PS13 isolate) supports the polyester‐degrading capability of this strain. Future studies will aim to purify and characterize these enzymes to determine whether they are functional and responsible for the polyesterase activity seen.

Here, we provide a system for improving plastic degrading capabilities of environmental bacterial communities to capitalize on a natural resource for the development of a biotechnological tool against plastic waste and identify *P. stutzeri* as an important environmental bacteria that has an understudied potential for plastic degradation.

## AUTHOR CONTRIBUTIONS

**Sophie Howard:** Data curation (equal); formal analysis (equal); investigation (equal); methodology (equal); supervision (equal); writing – original draft (equal); writing – review and editing (equal). **Clodagh Carr:** Data curation (equal); formal analysis (equal); investigation (equal); methodology (equal); writing – original draft (equal); writing – review and editing (equal). **Habteab Isaack Sbahtu:** Data curation (equal); writing – review and editing (equal). **Uche Onwukwe:** Data curation (equal); formal analysis (equal); writing – review and editing (equal). **Maria J. Lopez:** Formal analysis (equal); funding acquisition (equal); writing – review and editing (equal). **Alan Dobson:** Data curation (equal); formal analysis (equal). **Ronan McCarthy:** Conceptualization (equal); data curation (equal); formal analysis (equal); funding acquisition (equal); investigation (equal); methodology (equal).

## CONFLICT OF INTEREST STATEMENT

There are no conflicts of interest to declare.

## Supporting information


**Data S1.** Supporting Information

## Data Availability

All data is available upon reasonable request to the corresponding author. Sequence data has been deposited in the NCBI SRA database and GenBank and is available under the code PRJNA962804.
