# Supplementary material for: Enrichment of native plastic‐associated biofilm communities to enhance polyester degrading activity
Source: Environ Microbiol. 2023 Jul 28;25(12):2698–718. doi: 10.1111/1462-2920.16466 (PMC10947123; doi:10.1111/1462-2920.16466)
Supplement: Supplementary file 1 — Data S1. Supporting Information [file EMI-25-2698-s001.pdf]

Supplementary material

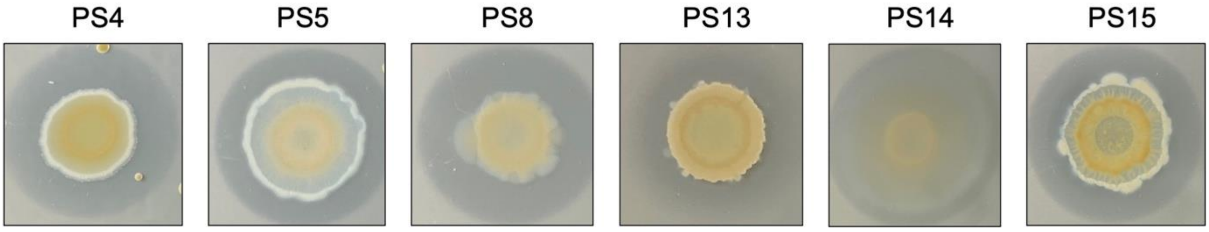

Figure S1: Different colony morphologies between isolates. (A) Single colony isolates of enriched strains found to be *P. stutzeri* from 16S rRNA sequencing, these have different colony morphology in colour, size and shape on 1% PCL agar plates, zones of clearance correspond to day 3 of Fig. 4A.

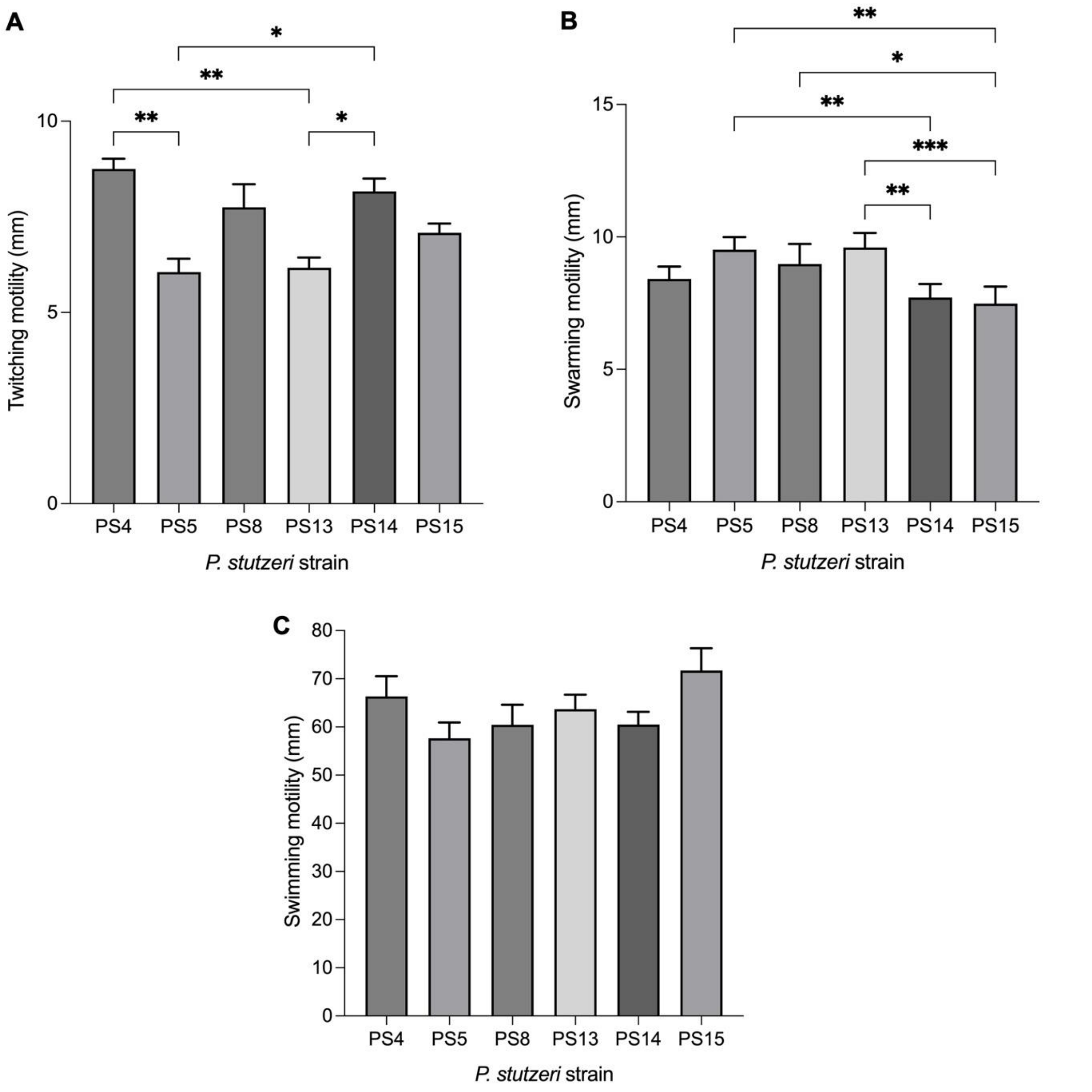

Figure S2: Motility assays of P. stutzeri isolates. (A) Twitching motility assay, mean and SD of 3 biological replicates, (B) swarming motility assay, mean and SD of 4 biological replicates, (C) swimming motility assay, mean and SD of 3 biological replicates. Each biological replicate consists of 3 technical replicates.

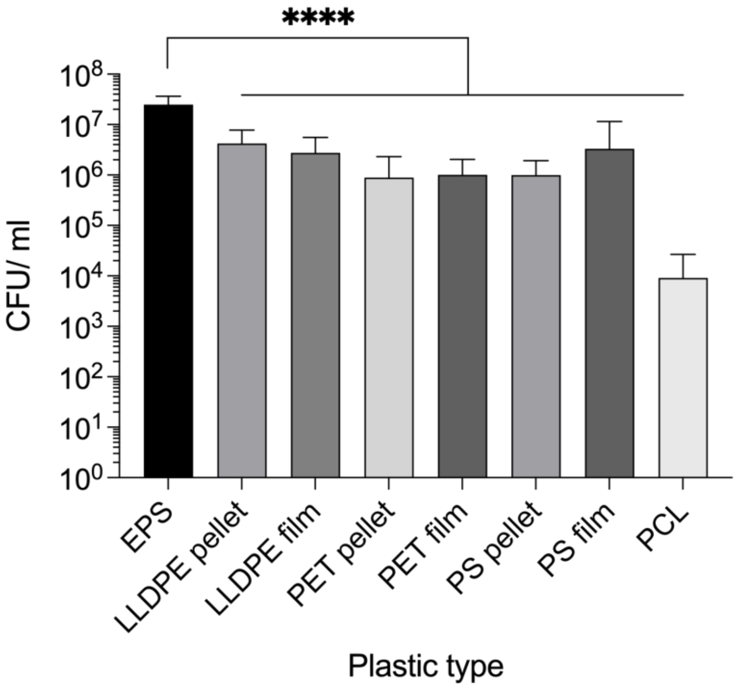

***Figure S3: P. stutzeri attachment to different plastics*.** PS13 single isolate *P. stutzeri* attached significantly more to EPS than the other plastics tested. Mean and SD from 9 pieces of plastic from 3 advanced technical repeats of 3 biological repeats (6 pieces of plastic from 2 biological repeats for PCL), one-way ANOVA was performed with multiple comparisons, the difference between EPS and each of the other plastics was $p<0.0001$.

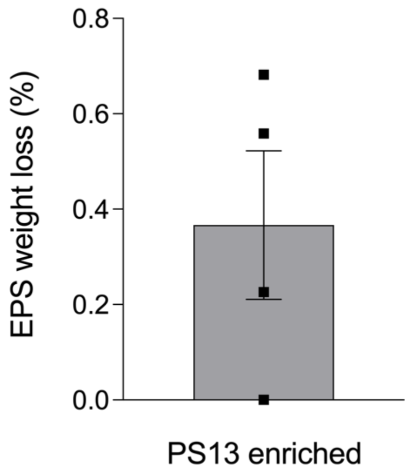

Figure S4: EPS weight loss assay. Overnight cultures of PS13 enriched communities were rinsed in PBS 3 times to remove nutrient rich media. OD_{600} 1 was inoculated into flasks of 15 mL of high-salt M9 minimal media with ~ 0.09 g of pure untreated EPS. Cultures were grown at room temperature with agitation for 77 days. The EPS was removed from the cultures, sonicated to remove attached bacteria, rinsed briefly in 2 % sodium dodecyl sulfate, rinsed with distilled water and dried before weighing. Mean and SEM of 4 advanced technical replicates. Whilst some weight loss was observed of the untreated EPS, due to the lightweight nature of EPS, the average weight loss of 0.37 % was only equivalent to less than 1 mg so was not deemed significant considering human and mechanical error with the weighing scales.

Table S1: Overall genome properties of PS13

Genome feature	PS13
**Size (bp)**	4610224
**Number of contigs**	12
**Largest contig (bp)**	1117968
**N50**	840153
**L50**	3
**GC content (%)**	62.92
**Completeness (%)**	100.0
**Contamination (%)**	0.14
**Number of coding sequences**	4291
**Number of hypothetical proteins**	1721
